# The prevalence of anxiety in pregnant women at Chris Hani Baragwanath Academic Hospital

**DOI:** 10.4102/sajpsychiatry.v30i0.2250

**Published:** 2024-07-23

**Authors:** Lisha Narayan, Corinne T. Johnson, Carina Y. Marsay

**Affiliations:** 1 Department of Psychiatry, Faculty of Health Sciences, University of the Witwatersrand, Johannesburg, South Africa

**Keywords:** antenatal, anxiety, GAD-7, maternal mental health, prevalence, pregnancy, psychiatry, associated factors

## Abstract

**Background:**

Non-psychotic mental disorders are common during the perinatal period. In South Africa, there are few studies on antenatal anxiety and these results vary. Antenatal anxiety does not only add to the burden of perinatal co-morbidity but has subsequent immediate and long-term effects on the mother, birth outcomes and her offspring.

**Aim:**

The aim of this study was to determine the prevalence of anxiety symptoms in pregnant women during the antenatal period and to determine associated factors.

**Setting:**

The study was conducted at an antenatal clinic located in Chris Hani Baragwanath Academic Hospital (CHBAH), Soweto, Johannesburg. Data were collected from March to December 2022.

**Methods:**

This was a prospective, cross-sectional study in which 200 pregnant women were interviewed. A biographical questionnaire and the generalised anxiety disorder questionnaire (GAD-7) were administered.

**Results:**

The prevalence of anxiety symptoms in pregnant women attending the antenatal clinic was 33%. Participants with anxiety were younger, employed and had lower perceived social support. Women with planned and wanted pregnancies had a lower prevalence of anxiety.

**Conclusion:**

One-third of the pregnant women screened positive for anxiety symptoms on the GAD-7. This is significantly higher compared to other studies carried out in the same facility previously. High-risk groups should be screened for anxiety.

**Contribution:**

This study prompts further studies and guiding policies on routine screening of pregnant women for anxiety and other mental illnesses during pregnancy.

## Introduction

Non-psychotic mental disorders are common morbidities in women during the perinatal period.^1^ Prior research has focused more on depression and the post-natal period. However, other disorders such as anxiety are shown to be increasingly evident or co-morbid during pregnancy. Anxiety-related disorders were found to be the most prevalent 12-month and lifetime disorders in the general population, with a prevalence of 15.8% according to the large-scale South African Stress and Health (SASH) study. Together with mood disorders, anxiety was more common among females.^2^ Pregnant women have been shown to experience higher levels of psychological distress compared with the general population in South Africa.^3^ During pregnancy, anxiety may either develop for the first time or pre-existing anxiety may become more severe.^4^

The presence of anxiety during pregnancy does not only add to the burden of perinatal co-morbidity but has subsequent immediate and long-term effects on the mother, birth outcomes and her offspring. Multiple mental health illnesses, such as depression and anxiety may be present co-morbidly during pregnancy or lead to the development of postpartum mental illness, thereby increasing the burden of disease on the mother in the perinatal period.^5,6^ About 30% to 58% of patients have been reported to meet criteria for co-morbid anxiety and depression.^4^

Maternal behaviour is affected by the presence of anxiety during pregnancy, with lower maternal cooperation, poor antenatal care attendance and increased maternal tobacco and alcohol use reported.^7^ A review study looking at the effects of anxiety and depression in pregnancy conducted by Alder et al. noted that the presence of anxiety in pregnancy was also associated with an increase in pregnancy symptoms such as nausea and vomiting, prolonged sick leave, more frequent visits to the physician, planned caesarean section delivery and increased use of analgesia during delivery.^8^ The experience of stress during pregnancy has been shown to affect birth outcomes and has been associated with the development of pre-eclampsia, lower infant birth weight, earlier delivery and smaller head circumference.^9,10,11,12^ Disruption in neurocognitive changes in the mother and maternal programming may reduce maternal responsiveness to infants, reduce nurturing and increase maltreatment of children.^13^

Emotional, behavioural and cognitive outcomes in children may also be affected. The underlying biological mechanisms are still being explored, but possible mediating factors have been identified.^14^ Identified effects during infancy and childhood include the presence of infant fear and distress; problems with attention regulation, adaption and behaviour; lower mental development scores; lower intellectual abilities and cognitive and language development; risk for emotional and behavioural problems and child mental health issues.^5,11,15,16,17,18,19^

These effects, however, are not inevitable, and better outcomes are seen in the absence of severe or chronic maternal mental disease, adversities and timeous management of perinatal mental illness.^13,20^ Simple integrated treatment of maternal mental illnesses, administered even by non-specialists, has shown to improve outcomes for both the mother and child in low and middle-income countries.^20^ Further local research into perinatal mental health disorders can help guide the development and implementation of effective national strategies, addressing this exceedingly important health issue. Thus, there is a need to add to the body of local research and evidence, which in turn can contribute towards guidelines on routine screening and management of common perinatal mental health illnesses in South Africa.

## Aim and objectives

The aim of this study was primarily to measure the prevalence of symptoms of anxiety in pregnant women attending the antenatal clinic at Chris Hani Baragwanath Academic Hospital (CHBAH). In addition, we examined the association between the presence of anxiety symptoms in pregnancy and maternal factors including socio-demographic characteristics, obstetric factors, psychiatric co-morbidity and medical co-morbidity.

## Research methods and design

### Study design

This was a prospective, cross-sectional, quantitative and observational study.

### Setting

The research was conducted at the CHBAH antenatal clinic. The hospital is a public, academic and tertiary-level institution situated in Soweto, Johannesburg.

### Study population and sampling strategy

A sample size of 200 participants was used, which was calculated based on a confidence level of 95%, a prevalence rate of 15.2%, and an estimated population size of 25460. Pregnant women were recruited from the antenatal clinic at the hospital between March and December 2022. A convenience sampling method was used. Prospective participants in the waiting area were given an information sheet detailing all the aspects of the study. Once participants agreed to engage in the study, they were screened to ascertain if they met the relevant inclusion and exclusion criteria, and a signed consent was obtained. The hospital where the study was conducted, being a tertiary academic institution, runs separate high-risk clinics for those with previous perinatal complications and physical health conditions. Participants were sampled on different days of the week, not only on high-risk clinic dates.

Participants who were excluded from the study included those who were under the age of 18, who have not had a first antenatal booking, had significant intellectual impairment, were attending the maternal mental health clinic or could not communicate proficiently in English.

### Data collection

A structured self-reported questionnaire was used to collect the relevant data. Data collection was carried out in an interview style, on a paper-based questionnaire and investigator administered in a private room within the clinic. Medical and obstetric information was additionally obtained from viewing the patient’s clinical record at the time of the interview, if required.

The first part of the questionnaire aimed to acquire data regarding socio-demographic characteristics, obstetric factors, and psychiatric and medical co-morbidity. These variables were assessed as categorical data, with binary and nominal options and continuous data for age and gestation. The generalised anxiety disorder 7-item scale (GAD-7) was used for the second part of the research questionnaire to screen for the presence of anxiety symptoms. It is one of the identified recommended tools to screen for anxiety during pregnancy.^21^ It consists of seven items with Likert-responses and relevant scoring between 0 and 3, with a total range of 0 to 21 points.^22^ A cut-off score of 10 or more has been recommended for the general population, with higher scores reflecting increased severity of symptoms and less than 10 indicating no need for treatment.^22^ Use of the GAD-7 is freely available.^23,24^ Participants with scores of 10 or more were referred to the maternal mental health clinic using a referral letter provided to the participant for further assessment and management.

### Data analysis

The data were captured on Microsoft Excel^TM^. All statistical analyses were conducted using R software (R version 3.4.2; https://www.r-project.org). Tests are two-tailed probability values and statistical significance is accepted when *α* ≤ 0.05. Data are presented in tables or figures, as appropriate. Continuous variables are reported as mean and standard deviation (s.d.). Categorical data are presented as counts and percentages. The association between categorical socio-demographic and clinical variables was analysed using Pearson’s Chi-squared tests or Fisher’s exact tests. The continuous variables, age of the patients and gestation were assessed for departures from normality using the Shapiro–Wilk test and subsequently analysed using a Mann–Whitney *U* test. Odds ratios are reported for significant Fisher’s *p* values.

### Ethical considerations

An informed, written consent was obtained from each participant prior to participation. Participant confidentiality was preserved, as no identifying data were noted on questionnaires; instead, participant numbers were allocated. Participants screening high for anxiety were given a referral for further assessment, with contact information for emergency care if required. Ethical approval from the ethics committee of the University of the Witwatersrand was obtained prior to conducting data collection. The ethics approval number is M210601. Permissions from the head of the department of the Obstetrics unit, Psychiatric unit and the Chief Executive Officer (CEO) at the facility were obtained. The research is also registered on the National Health Research Database (NHRD). The NHRD reference number is GP_202103_072. All procedures were in accordance with the ethical standards of the 1964 Helsinki Declaration and its later amendments.

## Results

**FIGURE 1:**
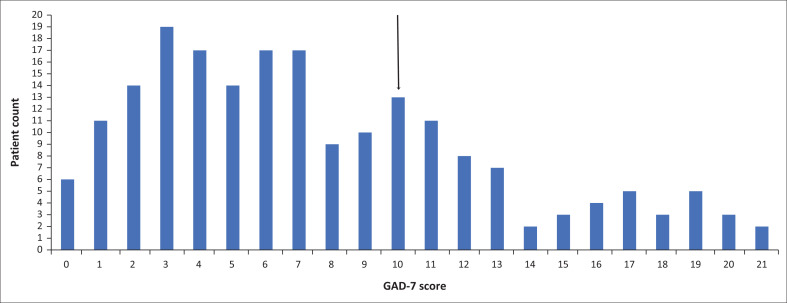
Frequency distribution of the generalised anxiety disorder 7-item scale scores of pregnant women at the antenatal clinic at Chris Hani Baragwanath Academic Hospital.

**FIGURE 2:**
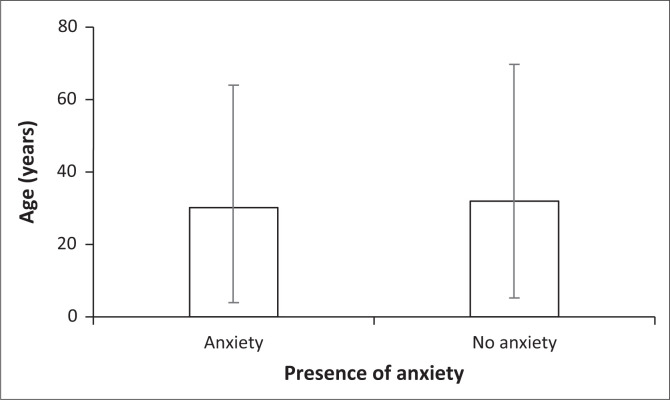
The age (median and interquartile range) of pregnant women with and without anxiety at the antenatal clinic at Chris Hani Baragwanath Academic Hospital.

This prospective study comprised a total of 200 patients at the antenatal clinic at CHBAH. The prevalence of a positive screen for anxiety on the GAD-7 was 33% (*n* = 66) (95% CI: 27% to 40%) (Figure 1). There was a significant association noted between patient age and anxiety scores (*Z* = 2.02, *p* = 0.0434). Patients classified with anxiety were significantly younger than those classified with no anxiety (Figure 2).

The level of education, being in a relationship, the relationship category and living with a partner were not associated with anxiety (Table 1). In contrast, employment and formal employment were associated with anxiety (Table 1). Patients who were not employed were 2.4 times (95% for OR 1.2 to 4.8) more likely to have no anxiety compared to those who were employed (Fisher’s exact test: *p* = 0.008). Similarly, patients who were not formally employed were 2.6 times (95% for OR 1.2 to 5.4) more likely to have no anxiety compared to those who had formal employment (Fisher’s exact test: *p* = 0.007).

**TABLE 1:** The association between socio-demographic variables and the presence of anxiety symptoms at the antenatal clinic at Chris Hani Baragwanath Academic Hospital (*N* = 200).

Variable	Anxiety		No anxiety		Statistics		
	Patient count (*n*)	%	Patient count (*n*)	%	*p*	*χ* ^2^	*df*
Level of education	-	-	-	-	0.702	0.71	2
Primary	7	25.9	20	74.1	-	-	-
Secondary	44	34.1	85	65.9	-	-	-
Tertiary	15	34.1	29	65.9	-	-	-
Employment	-	-	-	-	0.008†	-	-
Yes	27	47.4	30	52.6	-	-	-
No	39	27.3	104	72.7	-	-	-
Formal employment	-	-	-	-	0.007†	-	-
Yes	23	50.0	23	50.0	-	-	-
No	43	27.9	111	72.1	-	-	-
Relationship	-	-	-	-	1.000	-	-
Yes	60	33.0	122	67.0	-	-	-
No	6	33.3	12	66.7	-	-	-
Relationship category	-	-	-	-	0.573	1.12	2
Single	6	33.3	12	66.7	-	-	-
Partner	52	34.7	98	65.3	-	-	-
Married	8	25.0	24	75.0	-	-	-
Living with a partner	-	-	-	-	0.221	-	-
Yes	35	29.4	84	70.6	-	-	-
No	31	38.3	50	61.7	-	-	-

Note: Statistics = Fisher’s tests (*p*-values only) or Pearson’s Chi-square tests.

† , significant outcomes.

**FIGURE 3:**
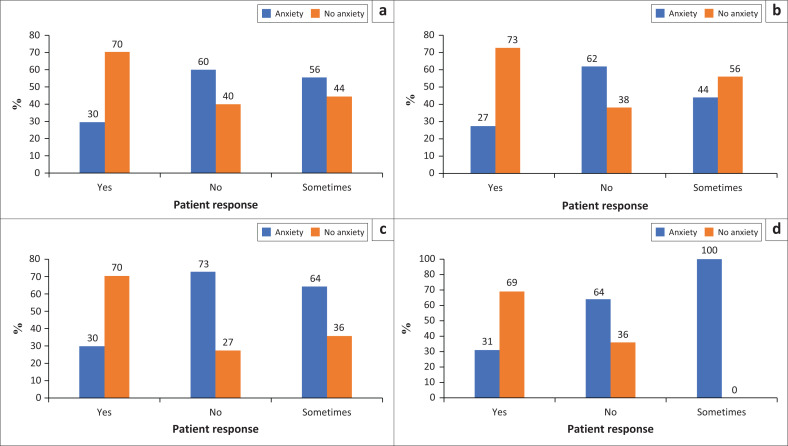
The association between perceived support and the presence of anxiety at the antenatal clinic at Chris Hani Baragwanath Academic Hospital: (a) Is there someone in your life you can talk to about your problems? (b) Is your partner or spouse supportive? (c) Are the people in your household supportive? (d) Is there someone to help you with childcare?

Patients were asked about their perceived social support from friends, family and their partners (Figure 3). Having someone to talk to increased the likelihood of no anxiety by 3.6 times (95% for OR 0.1 to 0.9) compared to having no one to talk to (Fisher’s exact test: *p* = 0.021). Having a supportive partner or spouse increased the likelihood of no anxiety by 4.9 times (95% for OR 0.1 to 0.7) compared to not having a supportive partner or spouse. Having supportive people in the household increased the likelihood of no anxiety by 2.6 times (95% for OR 0.03 to 0.7) compared to having no supportive people (Fisher’s exact test: *p* = 0.006). Having someone to help with childcare increased the likelihood of no anxiety by 2.2 times (95% for OR 0.1 to 0.7) compared to having no help (Fisher’s exact test: *p* = 0.002). There were no other significant differences.

The median gestation of patients with and without anxiety was 26 (interquartile range: 18.25 to 35.5) and 28 (interquartile range: 20 to 34.75) weeks, respectively. Gestation was not associated with anxiety scores (*Z* = 0.75, *p* = 0.453). Planned pregnancy was noted to be significantly associated with anxiety scores (Table 2). Having a planned pregnancy increased the likelihood of no anxiety by 2.1 times (95% for OR 0.2 to 0.9) compared to having an unplanned pregnancy (Fisher’s exact test: *p* = 0.030). Wanting the pregnancy was also significantly associated with anxiety scores (Table 2). Wanting the pregnancy increased the likelihood of no anxiety by 5.9 times (95% for OR 0.03 to 0.7) compared to not wanting the pregnancy (Fisher’s exact test: *p* = 0.007). Primigravity and previous and current pregnancy complications were not associated with anxiety (Table 2). Both psychiatric and medical co-morbidities were not associated with anxiety (Table 3).

**TABLE 2:** The association between obstetric variables and the presence of anxiety symptoms at the antenatal clinic at Chris Hani Baragwanath Academic Hospital.

Variable	Anxiety		No anxiety		*p*-value
	Patient count (*n*)	%	Patient count (*n*)	%	
**Planned pregnancy**	-	-	-	-	0.030†
Yes	18	23.4	59	76.6	-
No	48	39.0	75	61.0	-
**Wanted pregnancy**	-	-	-	-	0.007†
Yes	58	30.7	131	69.3	-
No	8	72.7	3	27.3	-
**Primigravida**	-	-	-	-	0.300
Yes	13	41.9	18	58.1	-
No	53	31.4	116	68.6	-
**Previous pregnancy complications**	-	-	-	-	0.648
Yes	30	35.3	55	64.7	-
No	36	31.3	79	68.7	-
**Current pregnancy complications**	-	-	-	-	0.088
Yes	31	40.8	45	59.2	-
No	35	28.2	89	71.8	-

Note: Statistics = Fisher’s tests (*p*-values only).

† , significant outcomes.

**TABLE 3:** The association between co-morbidities and the presence of anxiety symptoms at the antenatal clinic at Chris Hani Baragwanath Academic Hospital.

Variable	Anxiety		No anxiety		*p*-value
	Patient count (*n*)	%	Patient count (*n*)	%	
**Psychiatric history**	-	-	-	-	0.230
Yes	10	45.5	12	54.5	-
No	56	31.5	122	68.5	-
**Medical co-morbidities**	-	-	-	-	0.542
Yes	25	30.1	58	69.9	-
No	41	35.0	76	65.0	-

Note: Statistics = Fisher’s tests (*p*-values only).

## Discussion

### Key findings

In this study, we found the prevalence of anxiety symptoms in pregnant women attending the antenatal clinic at CHBAH to be 33%, based on scoring of 10 or more on the GAD-7. Participants with anxiety were younger, employed and had lower perceived social support. Women with planned and wanted pregnancies had a lower prevalence of anxiety. There was no noted significant correlation between anxiety and level of education, relationship status, gestation and psychiatric and medical co-morbidity.

### Discussion of key findings

Previous research has shown a varying pattern of the emergence and severity of anxiety prevalence in pregnancy. The prevalence of anxiety ascertained in this study is noted to be higher than that of previous studies conducted in the same facility. A study conducted by Redinger et al. at CHBAH between 2014 and 2016 noted a prevalence of 15.2% for anxiety in the first trimester on the State-Trait Anxiety Inventory.^6^ Using a clinical interview, a prevalence rate of 14.5% for antenatal anxiety disorders was reported in another study carried out at Rahima Moosa Hospital antenatal clinic in Johannesburg between July 2015 and April 2016.^25^ These prevalence rates are comparable with results found in other African, low-middle income countries at the time.^6^ Other studies reflected higher prevalence rates of antenatal anxiety. A cross-sectional study conducted at primary-level clinics in a low income setting in Cape Town between November 2011 and August 2012 revealed an overall prevalence for anxiety disorders in pregnancy of 23% using diagnostic interview techniques.^26^ This was comparable to another study, where high levels of psychological distress, 26.5%, were noted in pregnant women in Nkangala district in Mpumalanga using the Kessler Psychological Distress scale.^3^ A systematic review study looking at the prevalence of antenatal and postnatal anxiety in studies published up to January 2016 reported anxiety symptoms to have an overall prevalence of 22.9% across all three trimesters.^27^ Of note, in the above-mentioned studies, different methods of assessing anxiety were used, with some using different screening tools and others using clinical assessment. This may be attributed to the differences noted in prevalence.

South African studies utilising the GAD-7 as a tool to screen for anxiety in the antenatal period are noted to be limited at this time. A study carried out by Hoque et al. used the GAD-7 to assess the level of anxiety as one of the objectives of the study in pregnant women during the coronavirus disease 2019 (COVID-19) epidemic in South Africa. The study was conducted at a primary health care centre in Durban and found an anxiety prevalence of 35.7% using the GAD-7.^28^

The exposure to disease outbreaks may further exacerbate anxiety and negative emotional states during pregnancy.^29^ On 11 March 2020, the World Health Organization (WHO) declared the novel COVID-19 outbreak a global pandemic.^30^ Tomfohr-Madsen et al. conducted a rapid review and meta-analysis on depression and anxiety in pregnancy during COVID-19. A pooled prevalence of 25.6% was noted for depression and 30.5% for anxiety.^31^ This higher prevalence is in keeping with the prevalence noted in this study, and therefore, although data collection was conducted in 2022, the higher prevalence rate of anxiety may be an effect of the long-term repercussions of the COVID-19 pandemic. The WHO declared an end to COVID-19 as a public health emergency on 05 May 2023.^32^ In South Africa, the national state of disaster in relation to COVID-19 was lifted in April 2022.^33^

The finding of increased anxiety with younger age correlates with other studies.^6,34,35^ Lower level of education has been noted as a risk factor for anxiety in pregnancy; however, in this study, no significant correlation was noted between anxiety and level of education. A correlation was noted between employment, and specifically formal employment and higher levels of anxiety, which contrasts with other literature that notes unemployment as a risk for anxiety.^36^ However, a study conducted in Pakistan noted that mothers working outside the household had a higher prevalence of anxiety.^37^ The hospital in which this study was conducted services largely a lower socio-economic demographic, where often there is only one income sustaining multiple immediate and extended family members. Factors concerning employment and pregnancy such as maternity leave, childcare and meeting work demands may add to the burden of anxiety in pregnant working women.

Perceived social support was noted to increase the likelihood of not having significant anxiety. This is comparable to other studies. Lower perceived social support, high perceived stress, insufficient emotional and practical support, a history of abuse, adverse life events and having hostile in-laws have been noted to be associated with anxiety and other mental health conditions during pregnancy.^7,34,35,38,39^

Specific gestation and primigravity were not associated with anxiety in this study. There are inconsistent findings regarding the association between gravidity and the presence of antenatal anxiety.^40^ Some studies showed that being multigravida or having more children under the age of five in the household increased the risk of anxiety.^26,35^ Other studies linked primiparity and bearing the first child to be associated with increased anxiety.^34^

Having a planned and wanted pregnancy was associated with having no significant anxiety in this study. This was similar to other studies, where it was noted that maternal desire for pregnancy, including unplanned and unwanted pregnancies, were identified as predictors of anxiety.^3,26,35,36,38^

The presence of previous pregnancy complications and physical health conditions has been associated with anxiety during pregnancy.^7,39,40^ However, in this study, no significant association was noted.

Likewise in this study, there was no noted significant association between the history of past or present mental health illness and anxiety. This is a contradiction to other research that shows a history of mental illness to be a strong predictor of anxiety in pregnancy.^26,35,38,39^ However, this study sample excluded patients already attending the maternal mental health clinic and therefore may have excluded many participants with known mental health conditions at the time. We also did not explore if participants were on treatment or not for mental health conditions.

### Strengths and limitations

This study was conducted in a low-middle income (LMIC) setting, and detailed biographical data were collected. A higher prevalence of anxiety has been noted in this study. This may reflect the impact of the COVID-19 pandemic and warrants re-looking at antenatal anxiety prevalence.

The study population sampled was from a tertiary institution; therefore, it may represent a group with overall increased risk factors for anxiety, leading to the higher prevalence of anxiety that was noted in this study. However, this may highlight the importance of routine screening for anxiety and other mental health conditions in this high-risk population.

Teenagers were excluded from the sample population because of ethical concerns and issues surrounding consent. They however represent a high-risk group for mental illness during pregnancy. The interviews were conducted exclusively in English, thus excluding participants who were not fluent in the language.

The use of self-report measures may lead to biases, possibly inflated prevalence rates and it is based on the participant’s ability to recall information and cannot be verified. However, self-report measures, like the GAD-7, have practical relevance and utility as it can be used in primary health care settings and maternal clinics where clinical staff may not have expertise in psychiatric training.

Broad questions were used to assess previous and current pregnancy complications, psychiatric illness and medical illness. Asking specific questions may be more insightful regarding the specific details of these factors.

### Implications or recommendations

This being a cross-sectional study, it does not account for the emergence of anxiety symptoms that may possibly occur at different points of pregnancy. Further longitudinal studies exploring patterns of anxiety emergence, specific anxiety disorders, risk factors and the impact of anxiety disorders on obstetric outcomes may be beneficial. In addition, there is a need for studies that include a diverse group of participants, such as teenagers and individuals who are not proficient in English. These studies should also encompass different health care settings.

## Conclusion

This study highlights the high prevalence of anxiety in pregnant women and contributes to the body of research highlighting mental illness in the peri-partum period, thereby prompting further guidelines on routine screening of pregnant women, prioritising high-risk populations. Further longitudinal studies that investigate the prevalence patterns of anxiety during pregnancy, focussing on specific anxiety disorders, risk factors and including a more diverse range of participants, would provide valuable insights.
